# Feasibility study of cap-assisted endoscopic mucosal resection for gastric adenocarcinoma of fundic gland type

**DOI:** 10.1007/s10120-026-01762-7

**Published:** 2026-05-25

**Authors:** Risa Ashizaki, Tetsuya Yoshizaki, Yuta Higasa, Hiroshi Tanabe, Eri Nishikawa, Shinya Hoki, Ryosuke Ishida, Hitomi Hori, Tatsuya Nakai, Chise Ueda, Satoshi Urakami, Hirofumi Abe, Madoka Takao, Toshitatsu Takao, Masakyo Asahara, Yoshinori Morita, Takashi Toyonaga, Yuzo Kodama

**Affiliations:** 1 Division of Gastroenterology, Department of Internal Medicine, Kobe University Graduate School of Medicine, 7-5-1 Kusunoki-cho, Chuo-ku, Kobe, Hyogo 650-0017, Japan; 2 Department of Internal Medicine, Nishiwaki Municipal Hospital, Nishiwaki, Japan; 3 Department of Medical Device Engineering, Kobe University Graduate School of Medicine, Kobe, Japan; 4 Asahara Clinic, Akashi, Japan

**Keywords:** Cap-assisted endoscopic mucosal resection, Endoscopic submucosal dissection, Gastric adenocarcinoma of fundic gland type

## Abstract

**Background:**

Gastric adenocarcinoma of fundic gland type (GA-FG) is a rare subtype that typically arises in the upper stomach, making endoscopic submucosal dissection (ESD) technically challenging. Cap-assisted endoscopic mucosal resection (EMRC) is widely used for early gastric cancer due to its shorter procedure time and improved safety profile. We evaluated its efficacy and safety for GA-FG.

**Methods:**

We retrospectively analyzed patients with GA-FG who underwent EMRC or ESD between April 2013 and September 2024. Outcomes included en bloc, complete, and curative resection rates; procedure time; and adverse events. Odds ratios (ORs) were estimated using logistic regression, and inverse probability of treatment weighting was applied for adjustment.

**Results:**

We enrolled 70 patients with 77 lesions that were treated by EMRC (*n* = 57) or ESD (*n* = 20). En bloc resection was achieved in 100% of both groups. Complete resection rates were 96.5% for EMRC and 100% for ESD (*P* = 1.00). The EMRC group had significantly shorter median procedure time (6 vs. 46 min, *P* < 0.001) and fewer adverse events (OR 0.05, 95% CI 0.003–0.36; *P* = 0.002). No significant differences were found in curative resection (OR 3.47, 95% CI 0.86–14.08; *P* = 0.08) and intraoperative perforation (10% in the ESD group, 0% in the EMRC group; *P* = 0.06).

**Conclusions:**

Our findings suggest that EMRC is a safe and effective treatment for GA-FG, offering shorter procedure times and a lower risk of complications compared with ESD.

## Introduction

Gastric adenocarcinoma with chief cell differentiation was first reported by Tsukamoto et al. in 2007 [1]. In 2010, Ueyama et al. [2] proposed the concept of gastric adenocarcinoma of fundic gland type (GA-FG). Since then, GA-FG has gained recognition as a distinct histological subtype, and the number of reported cases and studies has gradually increased. Although GA-FG remains rare [3], its incidence appears to be rising, likely due to the decreasing prevalence of *Helicobacter pylori* infection. GA-FG is typically small, with an average size of less than 10 mm [4], and it exhibits a low frequency of vascular and lymphatic invasion [5–7], making endoscopic resection (ER) a reasonable treatment approach. However, GA-FG originates in the deep mucosal fundic gland region and can invade the submucosa even when small [8], necessitating careful consideration of treatment strategy.

Endoscopic submucosal dissection (ESD) is often the primary method of treatment for GA-FG due to its high histological complete resection rate and its ability to provide sufficient submucosal tissue for accurate assessment of lymphovascular and submucosal invasion. However, ESD can be technically challenging, particularly in cases where lesions are localized in anatomically difficult regions. Lesions on the upper third of the stomach are very difficult locations for performing ESD due to poor scope maneuverability and respiratory fluctuations, which often lead to prolonged procedure time and increased complication rates, including bleeding and perforation. Furthermore, a lesion located in the upper third of the stomach and longer procedure time are frequently reported risk factors for intraoperative perforation [9–16]. As GA-FG frequently arises in the upper third of the stomach [4], ESD inherently carries a high risk of complications.

Cap-assisted endoscopic mucosal resection (EMRC) offers a potentially safer and more efficient alternative for lesions in difficult locations. Compared with ESD, EMRC is associated with shorter procedure times and a lower risk of perforation. Among EMR techniques, EMRC is the most universal and least dependent on endoscopist skill variability. Despite these advantages, the efficacy and safety of EMRC for GA-FG remain to be elucidated. This study aimed to evaluate the effectiveness and safety of EMRC as a treatment option for GA-FG.

## Methods

### Patients and methods

This study was a retrospective observational study conducted at a single high-volume center in Japan. We included patients who received EMRC or ESD for GA-FG at Kobe University Hospital between April 2013 and September 2024. The inclusion criterion was histologically diagnosed GA-FG. The exclusion criteria were lesions with no confirmed tumor on histopathology after endoscopic treatment and duplicate lesions in the same resection specimen. Synchronous multiple lesions were simultaneously resected using EMRC or ESD.

This study was conducted in accordance with the Declaration of Helsinki and the Ethical Guidelines for Medical and Health Research Involving Human Subjects and was approved by the Ethics Committee of Kobe University School of Medicine (Approval number: B240201). Information about the study was made publicly available on the Internet, and participants were provided with the option to opt out.

### Modality selection for GA-FG

During the early adoption phase of EMRC at our institution, treatment selection between EMRC and ESD had not yet been fully standardized and was influenced by operator judgment and case-specific feasibility. Thereafter, ESD tended to be selected for lesions measuring ≥ 10 mm, closely adjacent synchronous multiple lesions requiring en bloc resection, and lesions in which gastric adenocarcinoma of fundic gland mucosa type was detected on preoperative biopsy. However, the final modality choice remained at the discretion of the operator, based on feasibility considerations such as complete cap capture on pre-suction testing and anticipated margin control. No formal institutional criteria for modality selection were defined.

### EMRC and ESD procedures

**Fig. 1:**
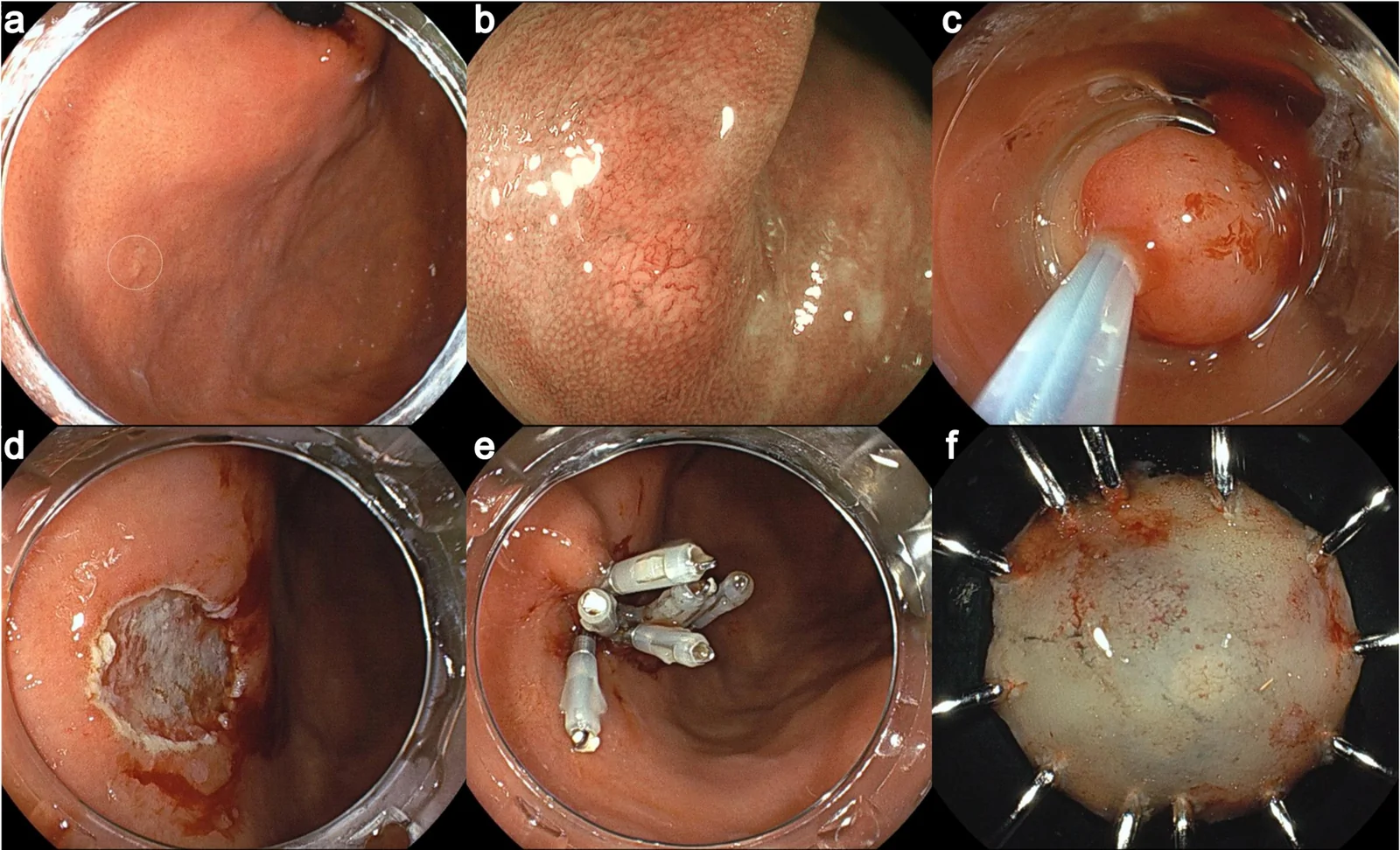
Cap-assisted endoscopic mucosal resection (EMRC) for a gastric adenocarcinoma of fundic gland type. **a** A flat, elevated, whitish lesion measuring 1.5 mm was located at the gastric fornix (white-dotted circle). **b** Narrow-band image. **c** The lesion is aspirated into the cap after submucosal saline injection and resected using a snare with coagulation current. **d** Mucosal defect after EMRC. The lesion is resected in a single piece without complication. **e** The wound is closed with endoclips. **f** Resected specimen

In both EMRC and ESD, a single-channel endoscope (EG-L580RD7; Fujifilm Corporation, Tokyo, Japan, GIF-H290T; Olympus Medical Co., Ltd, Tokyo, Japan) and an electrosurgical generator (VIO 300D or VIO3; ERBE Elektromedizin GmbH, Tübingen, Germany) were used. The details of the EMRC method are as follows [17]. A cap with a diameter of 13.8 mm (MAJ-290; Olympus) was fitted to the tip of the endoscope. Saline solution was injected into the submucosa beneath the lesion using an injection needle. An electrosurgical snare (SD-221 L-25; Olympus) was deployed within the cap, and the lesion was aspirated into the cap. The snare was then tightened, and a forced coagulation current was applied to achieve resection of the lesion. ESD was performed primarily with FlushKnife (Fujifilm Medical, Tokyo, Japan) or Dual Knife J (Olympus, Tokyo, Japan). Lesion localization was identified using narrow-band imaging magnification endoscopy, and marking dots were placed outside the margin of the lesion. Saline solution was injected into the submucosa beneath the lesion using an injection needle, followed by a circumferential mucosal incision outside the marking dots and submucosal dissection below the lesion (Fig. 1).

### Histologic evaluation and assessment of therapeutic efficacy

**Fig. 2:**
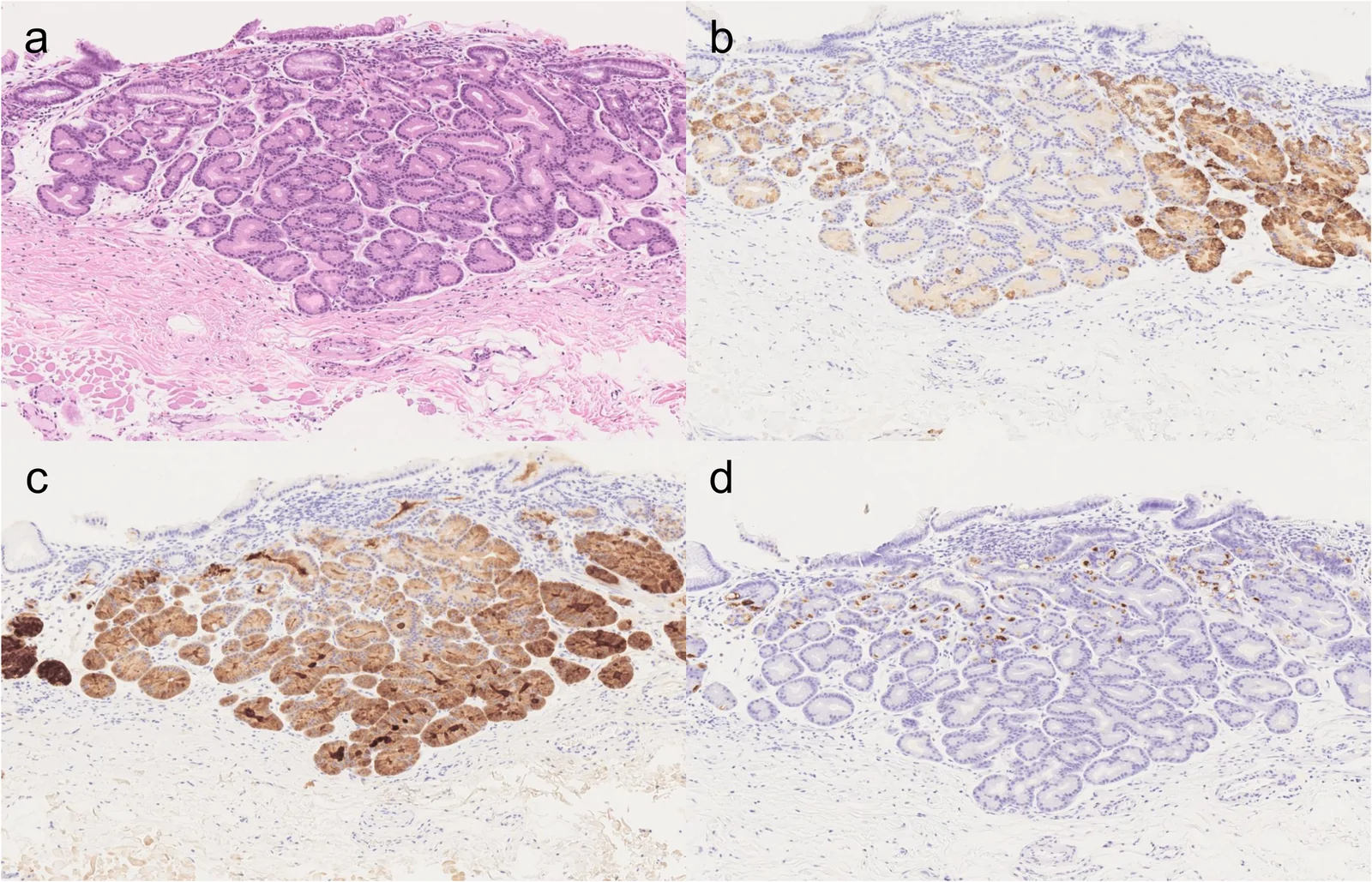
Histologic and immunohistochemical findings of the tumor shown in Fig. 1. **a** H&E staining (×10). The tumor shows compression of the muscularis mucosae with minimal submucosal invasion (10 μm). **b** MUC6 immunostaining (×10). Tumor cells show a mosaic pattern of weak to strong cytoplasmic positivity. **c** Pepsinogen-I immunostaining (×10). The tumor glands exhibit weaker staining compared with the surrounding fundic glands. **d** H⁺/K⁺-ATPase immunostaining (×10). Focal positivity is observed in tumor cells located in the superficial layer

Histologic assessment of resected specimens was performed according to the Japanese Gastric Cancer Treatment Guidelines 2021 (6th edition) [18]. A carcinoma that extended up to 500 μm below the lower border of the lamina muscularis mucosae was defined as SM1. Curative resection was defined as when a tumor was resected en bloc with a tumor-invasion depth of M to SM1, with no lymphovascular involvement and tumor-free margins. Resections that did not satisfy one or more of the criteria for curative resection were considered non-curative resections (Fig. 2).

### Definitions

En bloc resection refers to the resection of a lesion in one piece. Complete resection was defined as an en bloc resection with negative lateral and vertical tumor margins. For technical assessment of ER, the en bloc resection rates, complete resection rates, curative resection rates, resected specimen sizes, and procedure times were compared between EMRC and ESD. The resected specimen sizes and procedure times were obtained by referral to the relevant database or pictures taken during the procedure. The procedure times were measured from the initial injection to the total removal of the lesion. The resected specimen sizes were measured by the long diameter of the specimen.

### Complications

Bleeding related to the procedure was defined as bleeding that required blood transfusion or postoperative hemostatic treatment such as endoscopic clipping or thermocoagulation. Intraoperative perforation was defined as rupture of the serosa during ER. Delayed perforation was defined as sudden abdominal pain with intraperitoneal free gas on abdominal CT. Muscle layer damage was defined as a tear or thinning of the muscularis propria observed during the ER procedure, as indicated by endoscopic visualization of the damaged layer.

### Statistical analysis

The study dataset included patients with multiple lesions; however, for the purpose of data analysis, the various quantities observed in these patients were assumed to constitute statistically independent observations. Quantitative variables in this study were described as medians and interquartile range (IQR), whereas categorical variables were summarized by their counts (frequencies). The EMRC group was compared with the ESD group. Quantitative variables were compared using Mann–Whitney U test. Qualitative variables were compared using Fisher’s exact tests. A logistic regression model was used to calculate the odds ratio (OR) of curative resection and total adverse events. To adjust for confounding, we applied inverse probability of treatment weighting (IPTW) based on the propensity score, estimated with a logistic model including age, sex, lesion size, macroscopic shape, and tumor location. A *P* value < 0.05 was considered significant. All analyses were performed using JMP Pro 18 (SAS Institute Inc., Cary, NC, USA).

## Results

Between April 2013 and September 2024, 79 patients with 89 lesions underwent EMRC or ESD at Kobe University Hospital. A total of 12 lesions were excluded: nine lesions with no confirmed tumor on histopathology after endoscopic treatment, and three duplicate lesions identified in the same resection specimen. Finally, 70 patients with 77 lesions treated with EMRC or ESD were included. Characteristics of the patients and endoscopic features of the lesions are summarized in Table 1. Of the lesions, 34 (44.2%) occurred in male patients and 43 (55.8%) in female patients. The median age of the patients was 70 years (interquartile range [IQR]: 59–75). All lesions were detected during esophagogastroduodenoscopy by routine endoscopic screening or patients presenting with nonspecific abdominal symptoms. Sixty-five lesions (84.4%) were found in the upper third of the stomach, 12 (15.6%) in the middle third, and none (0%) in the lower third. Among the lesions in the upper third, 32 (49.2%) were observed in the fornix and nine (13.8%) in the cardia. There were no significant differences in patient characteristics and their lesions between the two groups. The ER results are shown in Table 2. No cases initially attempted with EMRC required conversion to ESD. Tumor size was significantly smaller in the EMRC group than in the ESD group (median: 4.0 mm, IQR: 3.0–5.0 mm vs. median: 6.5 mm, IQR: 4.0–9.3 mm, *P* = 0.001). The depth of invasion was M in 42 lesions (54.5%), SM1 in 27 lesions (35.1%), and SM2 or deeper in eight lesions (10.4%), with no significant difference between the two groups (*P* = 0.05). Venous invasion was present in one of 77 lesions (1.3%). No lymphatic invasion occurred.

**Table 1:** Baseline characteristics of patients and their lesions

	EMRC (*n* = 57)	ESD (*n* = 20)	*P* value
Age, years	70 (58–75)	69 (64.8–73)	0.94
Gender, male	22 (38.6%)	12 (60%)	0.12
Tumor location			0.28
Upper	50 (87.7%)	15 (75%)	
Middle	7 (12.3%)	5 (25%)	
Lower	0 (0%)	0 (0%)	
Tumor localization			0.74
Anterior wall	9 (15.8%)	3 (15%)	
Posterior wall	17 (29.8%)	4 (20%)	
Lesser curvature	8 (14%)	2 (10%)	
Greater curvature	23 (40.4%)	11 (55%)	
Macroscopic shape			0.08
0-IIa	30 (52.6%)	13 (65%)	
0-IIb	26 (45.6%)	5 (25%)	
0-IIc	1 (1.8%)	2 (10%)	
Antithrombotic therapy	4 (7.0%)	3 (15%)	0.37

ESD, endoscopic submucosal dissection; EMRC, cap-assisted endoscopic mucosal resection

**Table 2:** Short-term outcomes of each procedure

	EMRC (*n* = 57)	ESD (*n* = 20)	*P* value
Lesion size, mm, median (IQR)	4.0 (3.0–5.0)	6.5 (4.0-9.3)	0.001
Specimen size, mm, median (IQR)	15.0 (13.0–17.0)	28.5 (22.8–34.3)	< 0.001
Procedure time, min, median (IQR)	6 (4–8)	46 (34.5–70)	< 0.001
En bloc resection	57 (100%)	20 (100%)	NA
Complete resection	55 (96.5%)	20 (100%)	1.00
Curative resection	52 (91.2%)	15 (75%)	0.11
Depth			0.05
M	33 (57.9%)	9 (45%)	
SM1	21 (36.8%)	6 (30%)	
SM2 or deeper	3 (5.3%)	5 (25%)	
Lymphatic invasion	0 (0%)	0 (0%)	NA
Venous invasion	0 (0%)	1 (5%)	0.26
Complications			
Intraoperative/ Delayed perforation	0 (0%)	2 (10%)	0.06
Muscle layer damage	0 (0%)	3 (15%)	0.02
Delayed bleeding	1 (1.8%)	0 (0%)	1.00

ESD, endoscopic submucosal dissection; EMRC, cap-assisted endoscopic mucosal resection; IQR, interquartile range; M, mucosal cancer; SM, minute submucosal cancer (< 500 μm from the muscularis mucosa); SM2, deep submucosal cancer (≥ 500 μm from the muscularis mucosa); NA, not available

### Procedure outcome

Complete resection rates (EMRC: 96.5% vs. ESD: 100%; *P* = 1.00) and curative resection rates (EMRC: 91.2% vs. ESD: 75%; *P* = 0.11) were high in both groups. En bloc resection was achieved in all cases for both groups. Two patients who underwent EMRC had positive horizontal margins. In one patient, the lesion was confined to the mucosa with a positive horizontal margin. In the other patient, the lesion had invaded the submucosa (SM1; 350 μm) and had both positive vertical and horizontal margins. In this patient, local recurrence was observed 9 months after the initial treatment. This patient subsequently underwent ESD as additional treatment. The procedure time in EMRC was significantly shorter than in ESD (median: 6 min, IQR: 4–8 min vs. median: 46 min, IQR: 34.5–70 min, *P* < 0.001).

### Safety

The frequency of total adverse events was 7.8% in all patients. Intraoperative perforation occurred in two cases in ESD. No delayed perforation occurred in either group. Muscle layer damage occurred in three cases in ESD. There was no significant difference in the incidence of perforation between the EMRC and ESD groups (*P* = 0.06), whereas a significant difference in the incidence of muscle layer damage was found (*P* = 0.02). Delayed bleeding occurred in one case in the EMRC group. There was no significant difference in the incidences of delayed bleeding between the two groups (*P* = 1.00).

### ORs of endpoints

Table 3 shows the ORs of each endpoint for EMRC versus ESD analyzed using the logistic regression model. The OR for curative resection was not significantly different between the two groups (OR 3.47, 95% CI 0.86–14.08, *P* = 0.08). The OR for total adverse events was significantly lower in EMRC (OR 0.11, 95% CI 0.02–0.56, *P* = 0.008). These findings were unchanged after IPTW adjustment (curative resection: OR 1.13, 95% CI 0.27–3.35, *P* = 0.85; total adverse events: OR 0.10, 95% CI 0.04–0.28, *P* < 0.001).

**Table 3:** Odds ratios of endpoints for EMRC compared with ESD analyzed by univariate and IPTW using propensity score analysis

	Before adjustment		After IPTW	
	OR (95% CI)	*P* value	OR (95% CI)	*P *value
Curative resection	3.47 (0.86–14.08)	0.08	1.13 (0.27–3.35)	0.85
Total adverse events	0.05 (0.003–0.36)	0.002	0.10 (0.04–0.28)	< 0.001

OR, odds ratio; ESD, endoscopic submucosal dissection; EMRC, cap-assisted endoscopic mucosal resection; CI, confidence interval; IPTW, inverse probability of treatment weighting

## Discussion

This study represents one of the largest investigations to date on the effectiveness and safety of EMRC for GA-FG, providing robust evidence based on a substantial number of cases. En bloc resection was successfully achieved in all cases, suggesting that both EMRC and ESD are effective treatments. Importantly, there were no significant differences in the curative resection rates between EMRC and ESD, suggesting the therapeutic viability of EMRC. Moreover, EMRC exhibited a significantly shorter procedure time compared with ESD, as well as lower complication rates. These results strongly support EMRC as a practical and less invasive alternative to ESD for GA-FG.

No randomized controlled trials have directly compared treatment outcomes between EMR and ESD or between different EMR and ESD techniques for early gastric cancer; however, meta-analyses have reported that ESD achieves a higher en bloc resection rate than EMR [19–22]. Although the standard treatment for GA-FG has not been established yet, a large-scale review of GA-FG cases reported that 273 of 303 (90.1%) were treated with ESD or EMR, with ESD accounting for 71% [4]. A study of long-term follow-up outcomes (range, 0–107 months) reported that only one lesion experienced local recurrence after treatment [23], supporting the hypothesis that both ESD and EMR are suitable treatment options for GA-FG in most cases.

GA-FG predominantly occurs in the upper third portion of the stomach [4, 24–26]. In a previous study, 93 lesions (73.8%) were located in this region, with 38 lesions (30.2%) in the fornix, and 21 lesions (16.7%) in the cardia [23]. Notably, ESD for early gastric cancer located in the upper third, particularly in the fornix, is technically demanding due to anatomical constraints, such as limited maneuverability, the close proximity and thinness of the muscularis propria, and the vertical orientation of the lesion, all of which contribute to an increased risk of perforation. In the left lateral decubitus position, the submersion of the area further reduces visibility and respiratory fluctuations exacerbate technical difficulties. Consequently, the perforation rate in the upper third (22.6%) is significantly higher than in the middle (2.8%) and lower (3.2%) regions (*P* < 0.0005) [9]. In this study, intraoperative perforation occurred in two ESD cases; however, there were none in the EMRC group (10% vs. 0%, *P* = 0.06). These findings are consistent with those of Nakamoto et al. who reported similar perforation rates (ESD: 3/122 [2.5%] vs. EMR: 0/80 [0%], *P* = 0.41) [27]. Delayed perforation is generally considered a more serious complication than intraoperative perforation, due to muscle layer necrosis from excessive thermal injury [28]. In this study, muscle layer damage occurred in three ESD cases, showing a significant difference compared with EMRC (15% vs. 0%, *P* = 0.02). Bleeding requiring transfusion occurred in one EMRC case, with no significant difference between the two groups (1.8% vs. 0%, *P* = 1.00). Importantly, the overall OR for total adverse events was significantly lower in the EMRC group than in the ESD group, suggesting that EMRC offers a safer treatment alternative to ESD.

The EMRC technique devised by Inoue et al. [29] is recognized as a safe and effective treatment for small lesions in the esophagus and stomach [30]. In the present study, the 100% en bloc resection rate in the EMRC group is likely attributable to the small median tumor size of 4.0 mm. Given that the cap used in this study has an outer diameter of 13.8 mm (with an internal diameter of approximately 11 mm), lesions of this size can be easily and completely captured within the cap, ensuring high technical success. A previous study comparing the treatment outcomes of EMRC and ESD for superficial esophageal cancer reported a lower curative resection rate for EMRC than ESD; however, when limited to lesions ≤ 15 mm, there was no significant difference in the en bloc and curative resection rates between the two techniques [31]. Given that GA-FG is generally small [4], and our finding that 74 lesions (96.1%) were ≤ 10 mm, EMRC appears to be a highly suitable first-line treatment for the majority of GA-FG cases.

However, the applicability of EMRC to larger GA-FG lesions remains limited by the physical constraints of the cap. A previous study comparing underwater EMR and EMRC for superficial non-ampullary duodenal epithelial tumors reported that lesion size ≥ 10 mm was an independent risk factor for piecemeal resection, and that EMRC for lesions ≥ 15 mm should be considered selectively only when complete cap capture is clearly feasible [32]. Based on these findings and our results, we suggest that while EMRC is excellent for GA-FG < 10 mm, ESD should remain the gold standard for lesions exceeding 15 mm to ensure curative resection. For lesions in the 10–15 mm range, the success of EMRC depends heavily on endoscopic technique. For instance, it can be difficult to keep the lesion centered in the field of view. This is not an issue when the lesion can be approached directly. However, if the lesion is aspirated at a slightly tangential angle, the mucosa distal to the cap is often drawn in. To avoid this, the lesion should be aspirated slightly distal to its center, using a site near the cap as a fulcrum.

This study has several limitations that should be considered when interpreting the results. First, it was a single-center retrospective study with a limited sample size, as GA-FG is rare. Prospective, multicenter studies with larger sample sizes are needed to establish a standardized treatment strategy for GA-FG. Second, the tumor size was significantly smaller in the EMRC group than in the ESD group (median: 4.0 mm, IQR: 3.0–5.0 mm vs. median: 6.5 mm, IQR: 4.0–9.3 mm, *P* = 0.001), which may have contributed, at least in part, to the favorable outcome of en bloc resection in all cases in the EMRC group. Therefore, the generalizability of these findings to larger lesions is limited. Third, EMRC and ESD were performed during the same period without randomization, raising the possibility of selection bias. Fourth, long-term outcomes after treatment were not investigated. Further long-term follow-up studies are required to comprehensively evaluate the efficacy of EMRC.

In conclusion, our study suggests that EMRC is a safe and effective treatment option for GA-FG, offering shorter procedure times and a lower risk of complications than ESD.
